# Predictive Value of the CA-125 Elimination Rate Constant K (KELIM) in Predicting Progression-Free Survival and Overall Survival in Epithelial Ovarian Cancer

**DOI:** 10.3390/medicina61071250

**Published:** 2025-07-10

**Authors:** Necim Yalcin, Aysun Alci, Mustafa Gokkaya, Gulsum Ekin Sari, Tayfun Toptas, Isin Ureyen

**Affiliations:** Department of Gynecologic Oncology, Antalya Training and Research Hospital, Saglik Bilimleri University, 07100 Antalya, Turkey; aysun_alci@hotmail.com (A.A.); mugokkaya@gmail.com (M.G.); drekingulsum@gmail.com (G.E.S.); isin.ureyen@gmail.com (I.U.)

**Keywords:** epithelial ovarian cancer, interval debulking, overall survival, primary debulking, progression-free survival

## Abstract

*Background:* It is crucial to predict the response to chemotherapy and identify prognostic markers for recurrence and survival in patients with epithelial ovarian cancer (EOC), in order to effectively manage patient care. The CA-125 elimination rate constant K (KELIM) has recently been developed as a means of assessing the chemotherapy response and has been tested mainly in patients enrolled in randomized controlled trials. The objective of this study was to investigate whether the KELIM score is a prognostic marker for progression-free survival (PFS) and overall survival (OS) in EOC, utilizing its role in predicting the chemotherapy response in real-life settings. *Method:* Demographic, surgical, and survival data of patients with EOC operated on in Antalya Training and Research Hospital between January 2015 and December 2021 were obtained from the electronic gynecological oncology clinic database system and analyzed retrospectively. *Results:* A total of 102 patients with EOC were included; 30 patients (29.4%) had a KELIM score ≥ 1 and 72 (70.6%) patients had a KELIM score < 1. In the group with a KELIM score < 1, recurrence and refractory disease occurred in 49 patients, while it was 11 patients in the group with a KELIM score ≥ 1 (*p* = 0.004). PFS was 12 months and 32 months in the groups with KELIM scores of <1 and ≥1, respectively (*p* = 0.012). There was no difference between groups regarding OS (*p* = 0.139). In the whole group, KELIM score (<1 vs. ≥1) and type of surgery (IDS vs. PDS) were found to be independent prognostic factors for PFS (RR = 0.44; 95%CI: 0.22–0.88; *p* = 0.021 and RR = 2.97; 95%CI: 1.76–5.01; *p* < 0.001, respectively). *Conclusion:* We found that a favorable KELIM score was associated with better PFS in all groups of patients undergoing surgery for EOC in a real-life setting. With the increasing number of studies, the KELIM score will play an important role in providing better guidance to clinicians at the initial presentation of patients and in subsequent treatment planning.

## 1. Introduction

Ovarian cancer is the third most common gynecological cancer worldwide after endometrial and cervical cancer, causing 313,959 cases and 207,252 deaths annually. It is also the seventh most common cause of cancer death in women and the eighth leading cause of cancer death worldwide [1]. Because ovarian cancers are initially asymptomatic, 80% of patients are diagnosed at advanced stages and have a poor prognosis [2].

The standard treatment of ovarian cancer is debulking surgery and chemotherapy given in varying sequences, followed by a phase of maintenance therapy. Despite today’s advanced surgical techniques and effective chemotherapeutic agents, 95% of patients develop recurrence within 5 years [3]. It is known that the stage of the disease and achieving the goal of complete resection in surgery are important prognostic factors for survival. However, as recently recognized by the European Society for Medical Oncology (ESMO)–European Society of Gynaecological Oncology (ESGO) conference consensus, indicators of tumor chemosensitivity are needed to understand the prognostic impact of this parameter on the success of first-line medical and surgical treatment [4,5].

Epithelial ovarian cancers (EOCs) constitute 90% of all ovarian cancers, and CA-125 (Cancer Antigen-125), a serum marker, is detected to be high in the blood in 80–90% of patients with advanced stage epithelial ovarian cancer [6]. The role of assessing the rate of the decline in CA-125 during treatment in predicting the effectiveness of chemotherapy has been examined in many studies, but inconsistent results have been obtained [7,8,9,10]. The Gynecologic Cancer Intergroup (GCIG) adopted a chemotherapy response criterion based on the decrease in the percentage of CA-125, and a 50% decrease in the CA-125 level that could be sustained for at least 28 days was determined as treatment response [11]. However, Lee et al. and Morgan et al. recently reported using data from the CALYPSO Phase III trial and the ICON-8 Phase III trial that this CA-125 response criterion is a poor predictor of the effectiveness of chemotherapy in primary and recurrent ovarian cancer [12,13]. Recent studies have concluded that longitudinal evaluations of the CA-125 value using mathematical algorithms will provide more effective results in determining the chemotherapy response, both in first-line chemotherapy and in recurrence. In this context, the CA-125 elimination rate constant K (KELIM) score was developed, which measures the rate of decline in the CA-125 value during the first 100 days of chemotherapy and is obtained by using a minimum of three CA-125 values. The KELIM score is a modeled kinetic parameter that can be used to interpret CA-125 clearance independent of renal function. In this model, a higher KELIM score means a faster CA-125 elimination rate and a better chemotherapy response [10].

In the present study, we aimed to investigate whether the KELIM score is a prognostic marker that can be used for progression-free survival (PFS) and overall survival (OS) in EOC by using its role on predicting the chemotherapy response

## 2. Materials and Methods

### 2.1. Study Design and Participants

This retrospective study included patients with EOC who were treated at Antalya Training and Research Hospital between January 2015 and December 2021. The inclusion criteria for the study were as follows: (1) patients aged 18 years and over, (2) patients with any stage of EOC histologically proven by pathology, (3) patients receiving chemotherapy with a diagnosis of EOC and (4) patients with at least three CA-125 values in their medical records within 100 days following the administration of the first chemotherapy cycle. Exclusion criteria were (1) patients under 18 years of age, (2) patients with non-EOC, (3) patients previously diagnosed with any cancer, (4) patients who received chemotherapy or radiotherapy for any cancer, (5) patients with fewer than three Ca-125 values detected in their medical records within 100 days after the first chemotherapy cycle, and (6) patients who did not finish first-line chemotherapy. The study was approved by the ethics committee of Antalya Training and Research Hospital. The study was conducted following the Declaration of Helsinki and all subsequent amendments.

### 2.2. Follow-Up

Patients were followed every 3 months for the first 2 years after adjuvant treatment, every 6 months until the 5th year, and annually thereafter. A pelvic examination, complete blood count and blood chemistry, and abdominal ultrasonography were performed at each follow-up. A chest X-ray was performed annually or in case of clinical suspicion. Thoracic and/or abdominal computed tomography was used when clinically indicated. The Ca-125 level was used in follow-up when it was above normal limits at the time of diagnosis.

### 2.3. Clinical Data Collection

Data on demographic characteristics, intraoperative findings, surgical–pathological outcomes, completeness of cytoreduction based on postoperative residual disease (maximum cytoreduction without visible disease, optimal cytoreduction with residual disease < 1 cm, and suboptimal cytoreduction with residual disease ≥ 1 cm), patients’ treatments, recurrence, and survival were collected from the electronic gynecological oncology clinic database system, pathology reports, and surgical records. Patients were staged according to the 2014 International Federation of Gynecology and Obstetrics (FIGO) criteria.

### 2.4. Calculation of the KELIM Score

As shown in previous studies [4,5,10], at least three CA-125 serum measurements obtained within the first 100 days after the start of chemotherapy are required to calculate the KELIM score. The KELIM score was calculated using the online calculator available on the Biomarker Kinetics website (https://www.biomarker-kinetics.org, accessed on 6 July 2025) after entering three CA-125 measurements (before the 2nd, 3rd, or 4th cycle) obtained from patients’ files and the clinical database of patients who underwent primary debulking surgery (PDS) or interval debulking surgery (IDS) at sequential intervals. The KELIM score was analyzed as a continuous and binary index test for a positive result defined by a cut-off point of 1 (≥1) or higher.

After the KELIM score was calculated, the entire study population was compared in terms of recurrence, cytoreduction, response to primary treatment, PFS, and OS in two groups, namely KELIM score < 1 and KELIM score ≥ 1. At the same time, KELIM scores were compared in terms of recurrence, cytoreduction, response to primary treatment, PFS, and OS in a subgroup analysis of PDS and IDS groups.

The period from surgery to recurrence or the last visit was defined as PFS, and the period from surgery to death or the last visit was defined as OS. Follow-up time was evaluated as the time between the surgery and the time of patient’s last examination (death or the last visit).

### 2.5. Statistical Anaylses

Statistical data were analyzed with SPSS (Statistical Package for Social Sciences) software version 22 (SPSS Inc., Chicago, IL, USA). Descriptive statistics were presented using the median and range. The Mann–Whitney U test was used to compare medians. The Chi-square test or Fisher’s exact test, where appropriate, was used to compare proportions and percentages in different groups. Kaplan–Meier survival estimates were calculated for detecting survival for Stage III–IV disease. The possible factors were entered into Cox regression analysis to determine the independent effects on PFS and OS. Statistical significance was considered at *p* < 0.05.

## 3. Results

In total, 177 patients were operated on between 2015 and 2021 with a diagnosis of EOC. Seventy-four patients were excluded since the KELIM score could not be calculated due to the absence of data. One patient was excluded when she died of a cause other than cancer while taking chemotherapy. A total of 102 patients were analyzed in the study.

The median age of the patients was 58 years old (range: 31–82). The median Ca-125 level was 807 μ/mL (range: 28–3609). The median operative time was 300 min (range: 150–600). The median length of hospital stay was 11 days (range: 8–22). The median time between debulking surgery and chemotherapy was 30 days (range: 14–57).

The distribution of histotypes were high-grade serous in 80 patients (78.4%), low-grade serous in 9 (8.8%), clear cells in 7 (6.9%), mucinous in 5 (4.9%), and low-grade endometrioid in 1 (1.0%).

In total, 62 patients (60.8%) had PDS, while 40 had neoadjuvant chemotherapy. Ascites was present in 36 patients (35.3%). Peritoneal carcinomatosis and extra-abdominal disease were present in 64 (62.8%) and 32 patients (31.4%), respectively. Cytoreduction was maximal, optimal, and suboptimal in 78 (76.5%), 19 (18.6%), and 5 patients (4.9%), respectively. The stage of the disease was Stage I and II in 29 patients (28.5%), while it was Stage III and IV disease in 73 patients (71.6%). Maximal cytoreduction was achieved in 78 patients (76.5%) in the whole group. The KELİM score was <1 in 72 patients (70.6%), while it was ≥1 in 30 patients (29.4%). The median follow-up time was 54 months (range: 21–80). The main characteristics of the patients are shown in Table 1.

In the group with a KELİM score < 1, recurrence and refractory disease occurred in 49 patients (68.1%), while it was 11 patients (36.6%) in the group with a KELIM score ≥ 1 (*p* = 0.004). PFS was 12 months (0–97) and 32 months (2–106) in the groups with KELIM scores of <1 and ≥1, respectively (*p* = 0.012). There was no difference between groups regarding OS (*p* = 0.139). Furthermore, a statistically significant difference was identified between the groups with KELIM scores of <1 and ≥1 with respect to disease recurrence (*p* = 0.004). The comparison between the groups with KELIM scores of <1 and ≥1 in the whole study group is shown in detail in Table 2.

The groups with KELIM scores of <1 and ≥1 were also compared in the subgroups of patients who did and did not take neoadjuvant chemotherapy. In these subgroups of patients, there were no differences between the groups with KELIM scores of <1 and ≥1 regarding recurrence, cytoreduction results, disease status after primary therapy, PFS, and OS. Details are demonstrated in Table 3 and Table 4.

**Figure 1:**
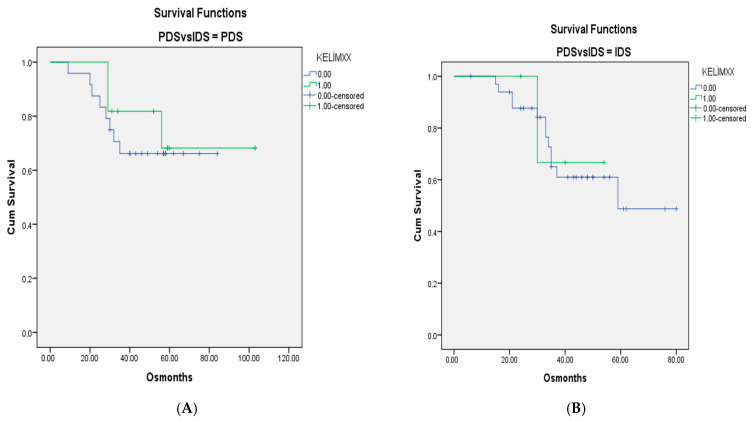
(**A**). OS curves for patients with Stage III–IV disease and KELIM scores of <1 and ≥1 in groups without neoadjuvant chemotherapy. (**B**). Survival curves for patients with KELIM scores of <1 and ≥1 in groups with neoadjuvant chemotherapy (*p* = 0.603).

**Figure 2:**
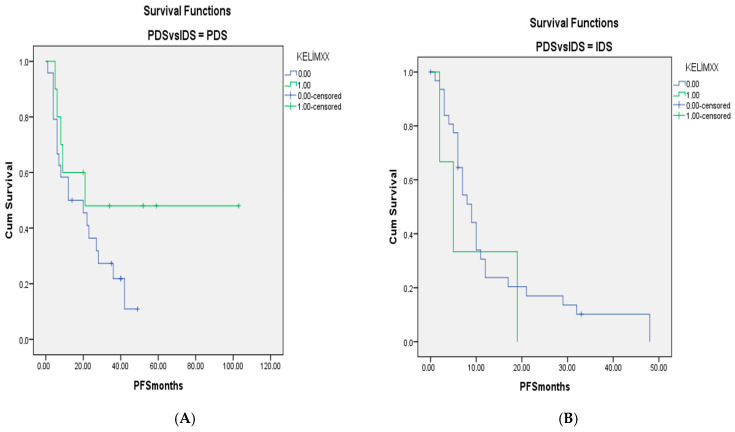
(**A**). PFS curves for patients with Stage III–IV disease and KELIM scores of <1 and ≥1 in groups without neoadjuvant chemotherapy. (**B**). Survival curves for patients with KELIM scores of <1 and ≥1 in groups with neoadjuvant chemotherapy (*p* = 0.440).

For when patients with only Stage III–IV disease were included, the survival curves for KELIM scores of <1 and ≥1 in the PDS-IDS groups are shown in Figure 1 and Figure 2. There were no differences between the groups (*p* = 0.603 and *p* = 0.440, respectively, for OS and PFS).

Cox regression analysis was performed for OS and PFS to determine the independent effects of the KELIM score and type of surgery (IDS vs. PDS) in the whole group. For OS the independent effects of neither the KELIM score nor the type of surgery could be shown. The KELIM score (<1 vs. ≥1) and type of surgery (IDS vs. PDS) were found to be independent prognostic factors for PFS (RR = 0.44; 95%CI: 0.22–0.88; *p* = 0.021 and RR = 2.97; 95%CI: 1.76–5.01; *p* < 0.001, respectively).

## 4. Discussion

EOCs are the most common cause of death among gynecological malignancies due to their asymptomatic course and diagnosis at advanced stages. Complete surgical resection and chemotherapy efficacy constitute the basis of treatment. Preoperative non-invasive methods to predict complete surgical resection and chemotherapy’s efficacy are needed, and recently, the KELIM score has been used for this purpose. In the present study, we aimed to investigate the effectiveness of the KELIM score in predicting survival retrospectively in patients operated on for EOC. In the present study, the KELIM score was found to be effective in predicting PFS, but not effective in predicting OS in the entire study population. However, when the PDS and IDS groups were analyzed separately in subgroup analyses, we found that the KELIM score was not effective in predicting PFS and OS in these groups.

In the present study, 30 patients (29.4%) had a KELIM score ≥ 1 and 72 (70.6%) patients had a KELIM score < 1. Although better results were obtained in all parameters subjected to statistical analysis in patients with a KELIM score ≥ 1, only recurrence rate and PFS were found to be statistically significant. In the study by Colomban et al. using data from three multicenter randomized controlled trials and that by Corbaux et al. using data from eight randomized controlled trials, the KELIM score was found to be an important independent prognostic factor for both PFS and OS in the first-line setting [14,15]. In the present study, although we obtained significant results for PFS in accordance with these studies, we could not reach significant results for OS, and we think that the most important reason for this is the low number of patients due to the inclusion of patients from a single center.

For patients diagnosed with ovarian cancer, reliable methods to predict the likelihood of recurrence following initial treatment are currently not available. Although the FIGO stage is a clinically meaningful tool for predicting 5-year survival, it is an unreliable predictor of the risk of recurrence. Despite the inclusion of clinical indicators such as age, performance status, blood markers, presence of comorbidities, tumor grade and histology, and residual tumor volume after surgery, the predictive value of these factors for 5-year survival remains insufficient to predict recurrence [16,17,18]. This has a limiting effect on clinicians’ ability to provide surveillance and survival support commensurate with the risks involved. In this context, the statistically significant finding of the KELIM score in predicting recurrence in this study will guide clinicians in planning and implementing follow-up care.

When the group of patients who underwent PDS was analyzed, the recurrence rate, the complete response rate after primary treatment, and the maximal cytoreduction rate were better in patients with a KELIM score ≥ 1 (Table 3), but none of these was statistically significant. However, when PFS and OS were analyzed, similar durations were obtained in patients with a KELIM score ≥ 1 and a KELIM score < 1. This may be due to the small number of patients in the PDS group because, if we perform a power analysis, there must be more than 70 patients in each group to show a 10% difference in survival rates between the groups with a KELIM score ≥ 1 and a KELIM score < 1. In addition, it may be due to the inclusion of Stage I and Stage II patients, in whom complete cytoreduction is relatively easier. While calculating the KELIM score of some patients who underwent PDS, we observed that the CA-125 value, which was previously high, tended to decrease after debulking, and then the CA-125 level decreased to normal limits after the first chemotherapy cycle was given. We believe that calculating the KELIM score according to this normal level may have prevented accurate results from being obtained in the PDS group. In the PDS group, we did not find any information in the literature regarding the inclusion of patients whose KELIM score was calculated with a CA-125 value that decreased to normal levels after debulking and the first chemotherapy cycle. Only Wagensveld et al. reported that patients with CA 125 < 35 IU/L were not included in their study [19]. When the literature is analyzed, there are only two studies evaluating the KELIM in patients with Stage I and Stage II EOC. The first study was conducted by Corbaux et al., and they found that a favorable KELIM score was associated with better PFS and OS in univariate and multivariate analyses in 628 Stage I and 515 Stage II epithelial ovarian cancer patients, which is inconsistent with the present study. The second study was conducted by Wagensveld et al. and because only four Stage II B and five Stage II C patients were included and most of the patients included in the study were in advanced stages (Stage III–IV), we think that more studies will be needed to evaluate the KELIM score in patients with Stage I and Stage II EOC [19,20].

In the present study, in the group of patients who underwent IDS after neoadjuvant chemotherapy, only 6 patients (15%) had a KELIM score ≥ 1 and 34 patients (85%) had a KELIM score < 1. The median PFS was 19 months and the median OS was 54 months in patients with a KELIM score ≥ 1, while the median PFS was 7.5 months and the median OS was 36 months in patients with a KELIM score < 1. Complete cytoreduction was achieved in 5 (83.6%) of 6 patients with a KELIM score ≥ 1 and in 20 (58.8%) of 34 patients with a KELIM score < 1 (Table 4). Although the complete resection rate, median PFS, and median OS months were better in patients with a KELIM score ≥ 1 (Table 4), this rate did not reach statistical significance. We think that the high numerical difference between the two groups may have prevented us from obtaining statistically significant results. In a study by You et al. using the CHIVA study on patients who underwent IDS after neoadjuvant treatment, in another study using data from the ICON-8 study, and in a study by Wagensveld et al., a favorable KELIM score was found to be an important independent prognostic factor for PFS and OS for patients who underwent IDS [19,21,22]. In recent single-center retrospective studies by Liontos et al., Piedimonte et al., and Zouzoulas et al. on serous ovarian cancer patients who underwent IDS after neoadjuvant chemotherapy, Liontos et al. and Piedimonte et al. found that a KELIM score ≥ 1 was associated with statistically increased PFS and OS, whereas in the study by Zouzoulas et al.,for a KELIM score ≥ 1, they could not find a statistically significant difference in OS and PFS in a multivariate analysis. In the study by Zouzoulas et al., the rate of patients with a KELIM score ≥ 1 was found to be 61.5%; in the study by Piedimonte et al., it was 35.9%; and in the study by Liontos et al., it was 43.4% [23,24,25]. In the present study, the low proportion of patients with a KELIM score ≥ 1 and the low number of patients who underwent IDS may have prevented us from obtaining meaningful results in terms of PFS and OS, and we think that in concordance with the present study, Zouzoulas et al. did not obtain meaningful results for PFS and OS due to the low number of patients.

When we look at the studies examining the relationship between the KELIM score and the probability of complete cytoreduction in the patient group undergoing IDS after neoadjuvant chemotherapy, Ducoulombier et al. found the KELIM score is an independent parameter associated with the probability of optimal cytoreduction, You et al. reported that a favorable KELIM value is an independent variable for the completeness of IDS, Bouvarel et al. found that the KELIM score is an independent and important determinant of the probability of complete surgery, and Zouzoulas et al. found a strong association between the KELIM score and residual disease in univariate and multivariate analyses [21,25,26,27]. However, Piedimonte et al. found no statistically significant difference in the rates of no gross residual disease between KELIM scores of <1 and ≥1. In the present study, although the rate of complete cytoreduction was found to be higher in patients with a KELIM score ≥ 1 in accordance with these studies, we did not reach statistical significance like Piedimonte et al., and we think it is due to the small number of patients [23].

## 5. Conclusions

The study is subject to certain limitations, such as its retrospective design and the presence of unaccounted-for biases that may have distorted the results. The relatively small number of patients included from a single center and the exclusion of those with Ca-125 values < 3 during chemotherapy have resulted in a relatively modest sample size. Furthermore, it should be noted that some of the subgroups (for example, the IDS group)also had small sample sizes. Finally, while the proportion of patients with a KELIM score < 1 was higher in this cohort, it should be noted that a KELIM score < 1 represents a higher-risk population. Considering the previously mentioned limitations, the present study has demonstrated a correlation between a positive KELIM score and superior PFS in all patient cohorts undergoing surgical intervention for EOC in a real-life setting. With the increasing number of studies examining the KELIM score in patients with EOC, determining whether the KELIM score is a sufficient biomarker for predicting the chemotherapy response will provide clinicians with better guidance during the initial examination of patients and play an important role in subsequent treatment planning, thereby contributing to clinicians’ ability to provide surveillance and survival support and predict survival outcomes.

## Figures and Tables

**Table 1:** Characteristic features of the patients.

Characteristics	Median (Range)		N (%)
Age (Years)	57 (31–82)		
Hospital Stay (Days)	11 (8–22)		
**Tumor Pathology**		High-grade serous	80 (78.4%)
		Clear cells	7 (6.9%)
		Low-grade serous	9 (8.8%)
		Low-grade endometrioid	1 (1.0%)
		Mucinous	5 (4.9%)
**FIGO Stage**		I–II	29 (28.4%)
		III–IV	73 (71.6%)
**Cytoreduction Surgery Performed**		Primary	62 (61.8%)
		Interval	40 (39.2%)
Residual Disease after IDS* and PDS*:		Maximal	78 (76.5%)
		Optimal (<1 cm)	19 (18.6%)
		Suboptimal (≥1 cm)	5 (4.9%)
**Clavien–Dindo Classification**		No	49 (48%)
		Grade 1	14 (13.7%)
		Grade 2	20 (19.6%)
		Grade 3	12 (11.8%)
		Grade 4	7 (6.9%)
		Grade 5	0 (0%)
Need for ICU* Admission		No	38 (37.3%)
		Yes	64 (62.7%)

Abbreviations: IDS*—interval debulking surgery; PDS*—primary debulking surgery; ICU*—intensive care unit.

**Table 2:** Comparison of clinical characteristics regarding the KELIM score in the whole group.

		Kelim Score		
		(*n*: 72) < 1	(*n*: 30) ≥ 1	*p*-Value
**Recurrence** ***n*** **(%)**	No	23 (31.9%)	19 (63.3%)	0.004
	Yes	30 (41.7%)	10 (33.3%)	
	Refractory Disease	19 (26.4%)	1 (3.3%)	
**Cytoreduction** ***n*** **(%)**	Maximal	52 (72.2%)	26 (86.7%)	0.288
	Optimal (<1 cm)	16 (22.2%)	3 (10%)	
	Suboptimal (≥1 cm)	4 (5.6%)	1 (3.3%)	
**Disease Status after Primary Therapy** ***n*** **(%)**	Complete Response	53 (73.6%)	29 (96.7%)	0.066
	Partial Response	16 (22.2%)	1 (3.3%)	
	Stable Disease	2 (2.8%)	0.(0%)	
PFS* (months)		12 (0–97)	32 (2–106)	0.012
OS* (months)		46 (6–97)	53 (24–106)	0.139

Abbreviations: PFS*—progression-free survival; OS*—overall survival.

**Table 3:** Comparison of clinical characteristics regarding the KELIM score in the group without neoadjuvant chemotherapy.

		Kelim Score		
		(*n*: 72) < 1	(*n*: 30) ≥ 1	*p*-Value
**Recurrence** ***n*** **(%)**	No	18 (47.4%)	16 (66.7%)	0.066
	Yes	13 (34.2%)	8 (33.3%)	
	Refractory Disease	7 (18.4%)	0 (0%)	
**Cytoreduction** ***n*** **(%)**	Maximal	32 (84.1%)	21 (87.5%)	0.844
	Optimal	3 (7.9%)	2 (8.3%)	
	Suboptimal	3 (7.9%)	1 (4.2%)	
**Disease Status after Primary Therapy** ***n*** **(%)**	Complete Response	31 (81.6%)	24 (100%)	0.083
	Partial Response	6 (15.8%)	0 (0%)	
	Stable Disease	1 (2.6%)	0 (0%)	
PFS* (months)		36 (1–97)	34 (5–103)	0.409
OS* (months)		50 (9–97)	52 (24–103)	0.691

Abbreviations: PFS*—progression-free survival; OS*—overall survival.

**Table 4:** Comparison of clinical characteristics regarding the KELIM score in the group with neoadjuvant chemotherapy.

		Kelim Score		
		(*n*: 72) < 1	(*n*: 30) ≥ 1	*p*-Value
**Recurrence** ***n*** **(%)**	No	5 (14.7%)	3 (50%)	0.134
	Yes	17 (50%)	2 (33.3%)	
	Refractory Disease	12 (35.3%)	1 (16.7%)	
**Cytoreduction** ***n*** **(%)**	Maximal	20 (58.8%)	5 (83.3%)	0.511
	Optimal	13 (38.2%)	1 (16.7%)	
	Suboptimal	1 (2.9%)	0 (%)	
**Disease Status after Primary Therapy** ***n*** **(%)**	Complete Response	22 (64.7%)	5 (83.3%)	0.821
	Partial Response	10 (29.4%)	1 (16.7%)	
	Stable Disease	2 (5.8%)	0 (0%)	
PFS* (months)		7.5 (0–48)	19 (2–106)	0.373
OS* (months)		36 (6–80)	54 (24–106)	0.353

Abbreviations: PFS*—progression-free survival; OS*—overall survival.

## Data Availability

Data are available upon reasonable request. Raw data were generated at Antalya Training and Research Hospital Health Science University. Derived data supporting the findings of this study are available from the corresponding author on request.
